# A Novel miRNA—hlo-miR-2—Serves as a Regulatory Factor That Controls Molting Events by Targeting CPR1 in *Haemaphysalis longicornis* Nymphs

**DOI:** 10.3389/fmicb.2020.01098

**Published:** 2020-05-29

**Authors:** Wen-Ge Liu, Jin Luo, Qiao-Yun Ren, Zhi-Qiang Qu, Han-Liang Lin, Xiao-Feng Xu, Jun Ni, Rong-Hai Xiao, Rong-Gui Chen, Muhammad Rashid, Ze-Gong Wu, Yang-Chun Tan, Xiao-Fei Qiu, Jian-Xun Luo, Hong Yin, Hui Wang, Zeng-Qi Yang, Sa Xiao, Guang-Yuan Liu

**Affiliations:** ^1^State Key Laboratory of Veterinary Etiological Biology, Key Laboratory of Veterinary Parasitology of Gansu Province, Lanzhou Veterinary Research Institute, Chinese Academy of Agricultural Sciences, Lanzhou, China; ^2^College of Veterinary Medicine, Northwest A&F University, Yangling, China; ^3^Xinjiang Animal Health Supervision Station, Ürümqi, China; ^4^Ruili Entry-Exit Inspection and Quarantine Bureau Inspection and Quarantine Comprehensive Technology Center, Yunnan, China; ^5^Ili Center of Animal Disease Control and Diagnosis, Ili, China; ^6^Jiangsu Co-innovation Center for Prevention and Control of Important Animal Infectious Diseases and Zoonoses, Yangzhou, China; ^7^Department of Engineering, Institute of Biomedical Engineering, University of Oxford, Oxford, United Kingdom

**Keywords:** *H. longicornis*, hlo-miR-2, cuticular protein, molting event, RNA interference

## Abstract

Successful completion of the molting process requires new epidermal growth and ecdysis of the old cuticle in *Haemaphysalis longicornis* (*H. longicornis*). MicroRNAs (miRNAs) participate in the development of organisms by inhibiting the expression of their target mRNAs. In this study, a novel tick-specific miRNA was identified and denoted hlo-miR-2 that serves as a novel regulator of molting events in *H. longicornis* nymphs by targeting a cuticular protein. The full length of this cuticular protein was first obtained and named it CPR1. A qRT-PCR analysis showed that hlo-miR-2 and CPR1 exhibit significant tissue and temporal specificity and that their transcription levels are negatively correlated during the molting process. CPR1, as a direct target of hlo-miR-2, was identified by a luciferase reporter assay *in vitro*. Agomir treatment indicated that the overexpression of hlo-miR-2 significantly reduced the protein expression level of CPR1, decreased the molting rate and delayed the molting time point in *H. longicornis* nymphs. RNA interference (RNAi) experiments demonstrated that CPR1 was significantly associated with the molting process in *H. longicornis* nymphs. Phenotypic rescue experiments convincingly showed that hlo-miR-2 participated in molting events by targeting CPR1 in *H. longicornis* nymphs. In summary, we present evidence demonstrating that miRNAs constitute a novel important regulator of molting events in addition to hormones. The described functional evidence implicating CPR1 in molting events contributes to an improved understanding of the distinct functions of the CPR family in ticks and will aid the development of a promising application of cuticular protein RNAi in tick control.

## Introduction

As a three-host tick, *H. longicornis* requires three hosts and undergoes two molting processes during its lifetime (Yatsenko and Shcherbata, 2014). The molting process, which can cause the generation of new epidermal growth and ecdysis of the old cuticle, is a prerequisite for the growth and development of *H. longicornis* ticks (Freitak et al., 2012; Zhou et al., 2013). Numerous external and internal stimuli, which might include seasonal changes, food intake, hormones and other factors, participate in the beginning of the molting process. The sharp increase in ecdysteroid (20-E) titers in the *H. longicornis* body during molting is consistent with the deposition of the new epicuticle and is an important factor in initiating the formation of the exuvial space (apolysis). A histological study of the tick cuticle ultrastructure has shown that the tick cuticle consists of two parts, the epicuticle and the procuticle, and the procuticle consists of two layers, namely, the inner and the outer endocuticle (Boulias and Horvitz, 2012; Asgari, 2013). Because cuticular proteins (CPs) are the main components of the arthropod cuticle, variations in the amounts and types of these proteins greatly affect the structure and properties of the arthropod cuticle (Choi and Hyun, 2012). Hence, CPs and CP-encoding genes can be used as relevant models for studying molting in ticks and the potential underlying molecular mechanisms. Furthermore, the molting process is very important in tick development, and interfering with the molting process of ticks will notably prevent or delay the development of ticks, resulting in the blockage of tick development and even death. Therefore, investigations of the effects of interference with the molting process will provide new ideas for the development of tick vaccines and the prevention and control of ticks.

MicroRNAs (miRNAs) are non-coding RNAs with a length of approximately 22 nt that posttranscriptionally regulate their target genes by binding to the 3′ untranslated regions (UTRs) of the corresponding mRNAs (Cenik et al., 2011; Fullaondo and Lee, 2012). Since the discovery of lin-4, this regulatory biological factor has been found in many organisms (Bejarano et al., 2010), such as mosquitoes, Drosophila, mites, ticks and other arthropods, and has been confirmed to be involved in the blood sucking, spawning, molting, and larval development processes (Caygill and Johnston, 2008; Huang et al., 2014; Hussain and Asgari, 2014; Kozomara and Griffiths-Jones, 2014; Zhang et al., 2015). A recent study on ticks revealed that miRNA-275 is involved in the physiological processes of blood digestion and ovary development and that miR-375 impacts oviposition and egg hatching in *H. longicornis* (Hao et al., 2017; Malik et al., 2019).

In our previous study, various miRNAs, including conserved and potentially novel miRNAs, were identified by deep sequencing of *H. longicornis* at different developmental stages. Among these miRNAs, a potential novel miRNA, named novel miRNA-2, exhibited high expression at the unengorged larval, unengorged nymph and unengorged adult developmental stages but not at the egg stage. Based on this finding, we speculated that novel miRNA-2 might play an important role in the maintenance of the developmental state of *H. longicornis*. Therefore, this study aimed to reveal the biological role of novel miRNA-2 in the development of *H. longicornis*.

## Materials and Methods

### Tick and Tissue Samples

Colonies of *H. longicornis* were established by the Lanzhou Veterinary Research Institute, Chinese Academy of Agricultural Sciences, Lanzhou, China (LVRI). The ticks were reared at 25°C in cages with 90% relative humidity and allowed to feed on the blood of New Zealand white rabbits. Under a stereoscopic microscope (Olympus, Japan), semi-saturated *H. longicornis* was dissected, and the tissues in the body cavity (salivary gland, midgut, ovary, and Malpighian tubules) were isolated and collected. Epidermal tissue was obtained by flushing with RNase-free water to remove the contents of the residual body cavity. A study conducted by Liu and Jiang (1998) found that the molting period of *H. longicornis* nymphs lasts 16.9 days, whereas our study revealed a duration of 15.47 days (Liu and Jiang, 1998). Therefore, we set the duration of the experimental observation in this study to 16 days. To more comprehensively and conveniently observe the dynamic changes during the molting period, we selected an experimental scheme involving the collection of samples every other day. The collected tissues were placed in the TRIzol reagent (Takara, Dalian, China) for the extraction of total RNA. The use of vertebrates in this study was approved by the Animal Ethics Committee of LVRI (Approval No. LVRIAEC2012-011).

### RNA Isolation and Quantitative Real-Time PCR (qRT-PCR)

Total RNA was extracted using the TRIzol reagent (Takara, Dalian, China). cDNAs of the mature sequence of novel miRNA-2 and the CP CPR1 (accession number MT274605) were produced using a PrimeScript^TM^ RT Reagent Kit (Takara) according to the manufacturer’s recommended protocol. All primer designs were performed using Primer Premier v5.0, and the primer details are shown in Table 1. qRT-PCR of miRNA and mRNA was performed using a SYBR^®^ *Premix EX Taq*^TM^ II Kit (Takara, Dalian, China) according to the manufacturer’s instructions. Each sample was measured in triplicate, and the relative expression levels were calculated using the 2^–ΔΔCt^ method and normalized to the expression of the housekeeping gene β-actin.

**TABLE 1:** Primers used in this study.

**Purpose**	**Primer name**	**Primer sequence (5′–3′)**
cDNA synthesis of miRNAs	Novel miRNA-2 SL primer	GTCGTATCCAGTGCAGGGTCCGAGG TATTCGCACTGGATACGACACTGTC
Real-time PCR analysis	Novel miRNA-2 F	CGGGCAAGAGAGCAATCCGT
	Novel miRNA-2 R	CAGTGCAGGGTCCGAGGTAT
	cuticular F1	ACAAGATCGCAATCCTCCTG
	Cuticular R1	TCCTGGTTGGCGTAGTTGAA
	β-actin F	CGTTCCTGGGTATGGAATCG
	β-actin R	TCCACGTCGCACTTCATGAT
Amplify the full length of the cuticular	GSP-F1	CAGCTTTCCTCTAAGGTCCGCTTCC
	GSP-R1	CATCTACAGGGGGGTGGATATGGAT
	GSP-F2	CGGACCCGAGGCCGCCATCGCCAAC
	GSP-R2	GGAAAGACGCTGTACGAGATCCCGC
Amplify the 3′ UTR of the cuticular	Cuticular F2	AGCTTTGTTTAAACCCTGCGTGTGATT CCGTGTA
	Cuticular R2	GCTCTAGAAAGAGGGAAGGAACGGG TAG
*In situ* hybridization	Cuticular probes	FAM-GGCAACGCAGGCGCAGGCCAGGAG GATT-FAM
	Novel miRNA-2 probes	CY3-ACTGTCCACGGATTGCTCTCTT

*SL means Stem loop. FAM and CY3 means green and red dye, respectively. Restriction sites are underlined.*

### Simulation of the Secondary Structure of the Novel miRNA-2 Precursor

RNAfold 2.4.13^1^ was used to simulate the secondary structure of the novel miRNA-2 precursor. The following running parameters of RNAfold were used for the simulation: -p, calculates the partition function and base pairing probability matrix in addition to the MFE structure; -d2, this previous check is ignored, and dangling energies are added to the bases adjacent to a helix on both sides in any case; and -noLP, produces structures without lonely pairs (helices of length 1). For partition function folding, the algorithm disallows pairs that can only occur in isolation, and other pairs might still occasionally occur as helices of length 1 (Mathews et al., 2004).

### Prediction and Amplification of the Full Length of the Target Gene

Three miRNA target prediction programs, namely, RNAhybrid^2^, PITA^3^ and miRanda^4^, were used to predict the target gene of novel miRNA-2. The rapid amplification of cDNA ends (RACE) technique was used to amplify the full length of the target gene. The two pairs of GSP primers were used to amplify the 3′ UTR and 5′ UTR of the CPR1 according to the RACE kit instructions (Takara, Dalian, China). All the primers were designed using Primer Premier v5.0, and the primer details are shown in Table 1.

### Luciferase Reporter Assay

293-T cells (cultured by LVRI) were grown in DMEM (Gibco, Waltham, MA, United States) supplemented with 10% fetal bovine serum (FBS) (Gibco, Waltham, MA, United States) and 1% 1x antibiotic-antimycotic (Gibco). Luciferase constructs were generated by inserting the 3′ UTR of the CPR1 gene into the pmirGLO vector (Promega, Madison, WI, United States). A pair of primers was used to amplify the 3′ UTR of CPR1 and were designed using Primer Premier v5.0. The primer details are shown in Table 1. One hundred micrograms of reconstructed pmirGLO vector was cotransfected with 50 nM synthetic novel miRNA-2 mimic (RiboBio, Guangzhou, China) or negative control (NC) (RiboBio, Guangzhou, China) into 293-T cells using Lipofectamine 3000 (Invitrogen, United States). Transfection with only the reconstructed pmirGLO vector was performed as a blank control treatment. Forty-eight hours after transfection, a dual luciferase reporter assay was performed using the Dual-Glo^®^ Luciferase Assay System (Promega) according to the manufacturer’s instructions. The firefly luciferase activity of the pmirGLO vector was measured for the normalization of Renilla luciferase activity. The treatments were performed in triplicate, and the transfections were repeated three times (Hao et al., 2017).

### Colocalization of Novel miRNA-2 and CPR1 by Fluorescence *in situ* Hybridization (FISH)

For the assessment of novel miRNA-2 and CPR1 colocalization, antisense RNA detection probes targeting novel miRNA-2 and the CPR1 gene were designed and labeled with the dual fluorophores Cy3 and FAM, respectively, at Servicebio Biotechnology Company (Wuhan, China). A scrambled sequence and the sense probe for the target gene were used as NCs. Two days after microinjection, the tick nymphs were dissected in cold PBS buffer, fixed overnight in 4% paraformaldehyde, and incubated overnight at 37°C for probe hybridization (Nuovo et al., 2009; Yang et al., 2016). The tick samples were washed in PBS containing 5% Triton X-100 (v/v) for 10 min and stained with DAPI G1012 (Servicebio, Wuhan, China) at room temperature for 8 min. The signals of CPR1 and novel miRNA-2 were detected using an Eclipse CI upright fluorescence microscope (Nikon, Japan). The probes for the miRNA and target genes are listed in Table 1.

### Agomir and Antagomir Treatment

Agomir (Ag), micrON^TM^ agomir negative control #22 (NC), antagomir (Ant), and micrOFF^TM^ Ant negative control #22 (NC) were synthesized by RiboBio Company (RiboBio, Guangzhou, China).

Five days after blood meal ingestion, *H. longicornis* nymphs were dosed at a site between the third and fourth legs. The experimental group was administered 800 μM novel miRNA-2 Ag or Ant in 0.2 μL of RNase-free water, the NC group was administered 800 μM micr*ON*^TM^ Ag or micr*OFF*^TM^ Ant in 0.2 μL of RNase-free water, and no injection was administered to the blank control group. Each group included 50 *H. longicornis* nymphs collected 5 days after blood meal ingestion.

Thirty ticks were used for observation of the molting timepoint and molting rate, and the rest were used to detect the expression of related genes and proteins. Two days after injection, total RNA and protein were extracted from the ticks using the TRIzol^TM^ reagent (Takara, Dalian, China) in accordance with the manufacturer’s instructions. Moreover, the number of successfully completed molts and the molting timepoints after microinjection were observed and recorded.

### RNAi and Rescue Experiments

The double-stranded siRNA targeting CPR1 (iCut) and the NC for iCut, which exhibited no significant sequence similarity to the *H. longicornis* sequence, was synthesized by RiboBio (Guangzhou, China). Five days after blood meal ingestion, *H. longicornis* nymphs were dosed at a site between the third and fourth legs: the experimental group was administered 800 μM iCut in 0.2 μL of RNase-free water, and NC group was administered 800 μM NC iCut in 0.2 μL of RNase-free water, and no injection was administered to the blank control group.

In the rescue experiments, the tick nymphs were divided into four groups and treated as follows: coinjection with 0.2 μL of an Ant/iCut mixture (800 μM Ant and 800 μM iCut), coinjection with 0.2 μL of an Ant/NC iCut mixture (800 μM Ant and 800 μM NC iCut), coinjection with 0.2 μL of an NC Ant/iCut mixture (800 μM NC Ant and 800 μM iCut), and coinjection with 0.2 μL of an NC Ant/NC iCut mixture (800 μM NC Ant and 800 μM NC iCut).

Each group included 50 *H. longicornis* nymphs obtained 5 days after blood meal ingestion. Thirty ticks were used for observation of the molting timepoint and molting rate, and the rest were used for the detection of related genes and proteins. Two days after injection, total RNA and protein from the ticks were extracted using the TRIzol^TM^ reagent (Takara, Dalian, China) in accordance with the manufacturer’s instructions. Moreover, the number of successfully completed molts and the molting timepoints after microinjection were observed and recorded.

### Western Blot Analysis

Total protein from the ticks was extracted using the TRIzol^TM^ reagent (Takara, Dalian, China) according to the manufacturer’s instructions, separated on 12% SDS–PAGE gels and transferred to PVDF membranes (Millipore, United States). The membranes were then blocked in TBST containing 5% skim milk for 1 h at room temperature. For the detection of CPR1, the membranes were incubated overnight at 4°C with monoclonal antibodies against CPR1 at a 1:300 dilution and then incubated with a 1:5000 dilution of an anti-rabbit HRP-conjugated secondary antibody (Solarbio, Beijing, China) at room temperature for 1 h. CPR1 expression was detected by chemiluminescence (Thermo Fisher Scientific, United States).

### Scanning Electron Microscopy

The samples were fixed in 3% glutaraldehyde at room temperature for 2 h and transferred to 4°C for storage. The samples were fixed in 1% osmium tetroxide for 2 h, dehydrated in an alcohol gradient (30–50–70–80–90–95–100–100% alcohol), and incubated with isoamyl acetate for 15 min. The samples were then dried in a K850 critical point dryer (Quorum, United Kingdom) and subjected to gold sputtering for approximately 30 s with an MSP-2S instrument (IXRF, United States). Observations and imaging were performed under an SU8100 scanning electron microscope (HITACHI, Japan) (Liu et al., 2001).

### Statistical Analysis

All the data were analyzed using GraphPad Prism 7, and the results are presented as the means ± SEMs. The F test was used for equal variance detection, and the means were compared using Student’s *t*-test or the Wilcoxon test based on the following significance levels: ^∗^*P* < 0.05, ^∗∗^*P* < 0.01, ^∗∗∗^*P* < 0.001, and ^****^P < 0.0001.

## Results

### Characterization and Validation of Novel miRNA-2

**FIGURE 1:**
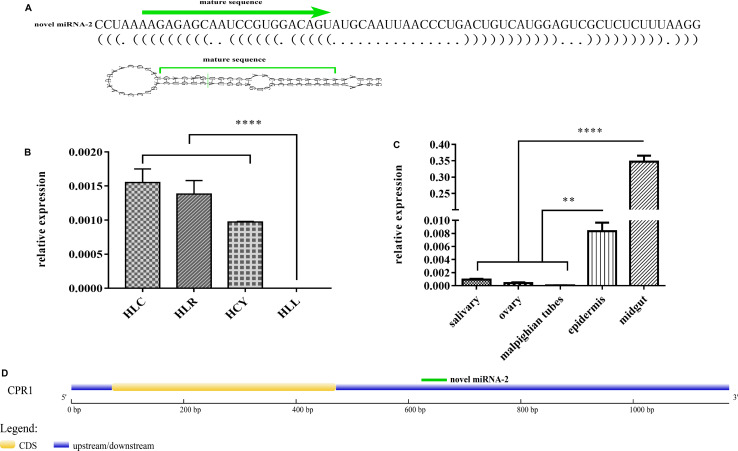
Characterization, expression patterns and target gene prediction of novel miRNA-2. **(A)** The novel miRNA-2 precursor is 69 nt in length with a stem-loop structure and the mature miRNA is 22 nt in length. **(B)** The relative expression levels of novel miRNA-2 in eggs, unengorged larvae, unengorged nymphs and unengorged adults were analyzed by qRT-PCR. HLL, HLY, HLR, and HLC are representative of eggs, unengorged larvae, unengorged nymphs, and unengorged adults of *H. longicornis*, respectively. **(C)** The relative expression levels of novel miRNA-2 in the salivary gland, midgut, ovarian, Malpighian tubules, and epidermal tissues were analyzed by qRT-PCR. **(D)** Structure of the CPR1 gene and location of the novel miRNA-2-binding site. All the data represent the results from three biological replicates, each of which included three technical replicates, and are presented as the means ± SEMs. **P < 0.01 and ****P < 0.0001.

The potential novel miRNA-2 needs to be further characterized and validated. One of the most notable characteristics defining a miRNA gene is a precursor with a typical stem-loop structure, and at most four symmetrical mismatches within the duplex and at most two asymmetric bulges of up to 2 bases are allowed (Ambros et al., 2003). Close scrutiny of the secondary structure of novel miRNA-2 precursor indicated that its stem-loop structure conformed to the established standards (Figure 1A).

### Tissue-Specific and Developmental Expression Patterns of Novel miRNA-2

Novel miRNA-2 was highly expressed in unengorged larvae, unengorged nymphs and unengorged adults but was not found at the egg stage by qRT-PCR analysis (Figure 1B). To determine the tissue expression patterns of novel miRNA-2, we performed a RT-PCR analysis of the five tissues of *H. longicornis*. The results demonstrated that novel miRNA-2 was abundantly expressed in both the midgut and epidermis but was expressed at low levels in the other tissues (salivary gland, ovary, and Malpighian tubules) (Figure 1C).

### Prediction of a Novel miRNA-2 Target Gene and Luciferase Reporter Assay-Based Identification of the Binding Site of Novel miRNA-2 on the Target Gene *in vitro*

Three publicly available bioinformatic tools for miRNA target gene prediction were used to predict the target gene of novel miRNA-2. The same prediction results obtained from all of the tools showed that the CPR1 gene contains a binding site for novel miRNA-2. We obtained the full-length sequence of the CPR1 gene using RACE technology. The full length of the CPR1 gene contained 1171 bp, and the coding sequence (CDS) was 339 bp in length (Supplementary Data 1). The putative novel miRNA-2-binding site in CPR1 is located at position 661, which is located at the 3′ UTR of CPR1, and this binding is a classical binding mode (Figure 1D).

**FIGURE 2:**
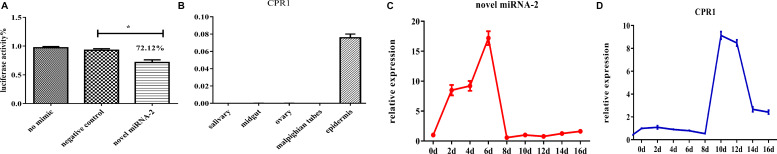
The target gene identification and expression patterns analysis of novel miRNA-2 and CPR1. **(A)** Dual luciferase reporter assay of CPR1. The data indicate the percent activity and are presented as the means ± SEMs from triplicate samples. The percentages are shown. *P < 0.05. **(B)** The relative expression levels of CPR1 in salivary gland, midgut, ovarian, Malpighian tubules, and epidermal tissues were analyzed by qRT-PCR. **(C)** The relative expression level of novel miRNA-2 in the epidermis was analyzed every two days (D: day) during the molting process of nymphs by qRT-PCR. **(D)** The relative expression level of CPR1 in the epidermis was analyzed every two days (D: day) during the molting process of nymphs by qRT-PCR. All the data are presented as the results from three biological replicates, each of which included three technical replicates, and are presented as the means ± SEMs.

The 3′ UTR of the CPR1 gene was inserted into the pmirGLO vector and cotransfected into 293-T cells along with a novel miRNA-2 mimic, whereas NC mimic/novel miRNA-2 and novel miRNA-2 were transfected alone as negative and blank controls, respectively. The fluorescence results showed that the luciferase activity of the pmirGLO- CPR1/novel miRNA-2 group was 72.12% of the values found for the NC and blank control groups, which indicated that CPR1 was a target gene of novel miRNA-2 *in vitro* (Figure 2A).

### Tissue-Specific Expression Patterns of CPR1

To assess the expression of CPR1 in different tissues, we performed a RT-PCR analysis of the five tissues of *H. longicornis*. As shown in Figure 2B, CPR1 was abundantly expressed in the epidermis but exhibited low expression in the other tissues (midgut, salivary gland, ovary, and Malpighian tubules).

### Detection of the Dynamic Expression of Novel miRNA-2 and CPR1 During the Molting Process of *H. longicornis* Nymphs by qRT-PCR

To compare the expression levels of novel miRNA-2 and CPR1, we performed a thorough time-course assay of these miRNAs in *H. longicornis* nymphs during the molting process by qRT-PCR analysis. Eight time points were selected for the collection of RNA samples from *H. longicornis* nymphs: 2, 4, 6, 8, 10, 12, 14, and 16 days post blood meal ingestion. The expression level of novel miRNA-2 in *H. longicornis* nymphs gradually increased from day 0 to 6 post blood meal ingestion, reached a peak on day 6, decreased sharply starting on day 8 and remained at a low level until the end of the molting process. Interestingly, CPR1 exhibited a lower expression level from day 0 to 8 post blood meal ingestion, and its level then increased rapidly beginning on day 10 and remained high until the end of the molting process (Figures 2C,D).

### Colocalization of the Novel miRNA-2 and Its Target Gene CPR1

**FIGURE 3:**
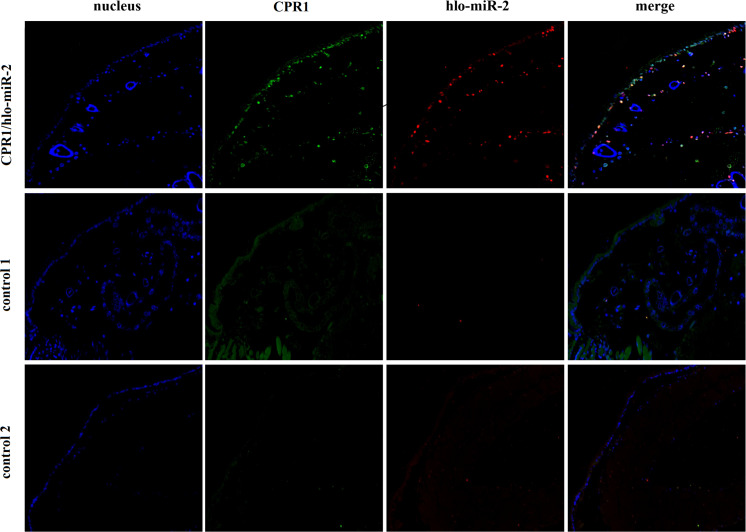
Colocalization of novel miRNA-2 with the target gene CPR1 in epidermal cells of ticks. The nucleic acid probe targeting novel miRNA-2 was conjugated to the dual fluorophore Cy3 (red), whereas the nucleic acid probe for the target gene CPR1 was conjugated to the dual fluorophore FAM (green). Probes targeting a scrambled nucleotide sequence were used as the control for miRNA, and probes targeting the sense nucleotide sequence were used as the control for the target gene. The overlapping of green (CPR1) and red signals (novel miRNA-2) is indicated by a yellow signal, which indicated the colocalization of novel miRNA-2 and its target gene CPR1. Control 1, scrambled miRNA and CPR1 antisense probes; Control 2, novel miRNA-2 and CPR1 sense probes. The images were acquired using an Eclipse CI upright fluorescence microscope (Nikon).

To determine whether novel miRNA-2 and CPR1 were colocalized in the epidermal tissue of tick nymphs, we performed *in situ* analyses of novel miRNA-2 and its target CPR1 via miRNA/mRNA fluorescence *in situ* hybridization. Both novel miRNA-2 and CPR1 were widely detected in the epidermal cells of ticks (Figure 3), which suggested that in the tick integument, CPR1 interacts directly with novel miRNA-2 in a spatially dependent manner.

### Novel miRNA-2 Overexpression Results in Severe Molting Defects

To determine the function of novel miRNA-2 in molting events, we overexpressed novel miRNA-2 by microinjecting Ag into *H. longicornis* nymphs at 5 days post blood meal ingestion. The transcript and protein levels of CPR1 were assessed on day 2 after microinjection. The qRT-PCR results showed that the CPR1 mRNA level was not significantly reduced in the Ag-treated group (0.956 ± 0.03445 fold), and as expected, the expression in the NC and blank control groups was unaffected. Moreover, the Western blot results showed that the expression of CPR1 in the Ag-treated group was lower than that in the NC (0.989 ± 0.05615 fold) and blank control (0.979 ± 0.06109 fold) groups, which suggested that overexpression of novel miRNA-2 can downregulate CPR1 expression.

**FIGURE 4:**
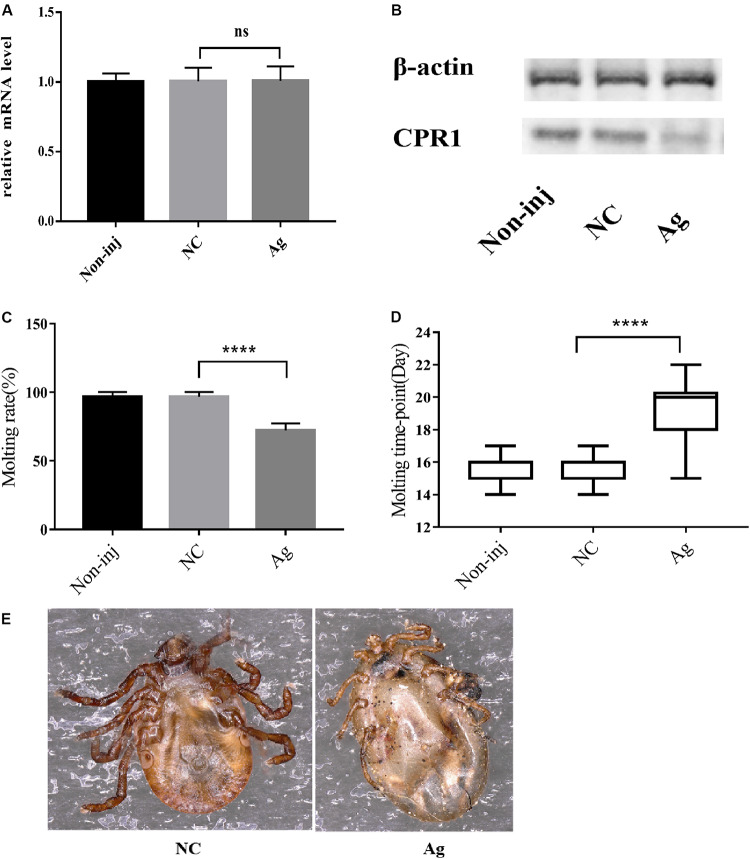
The overexpression of novel miRNA-2 significantly disrupted protein accumulation and delayed the molting time with increasing variation. **(A)** Transcription level of the target gene CPR1 after novel miRNA-2 overexpression. **(B)** Protein level of the target gene CPR1 after novel miRNA-2 overexpression. **(C)** Molting rate of nymphs injected with a novel miRNA-2 agomir. **(D)** Molting timepoint of nymphs injected with a novel miRNA-2 agomir. The data are shown as the means ± SEMs and were evaluated by Student’s *t*-test. ****P < 0.0001. **(E)** Representative phenotypes after feeding with novel miRNA-2 agomir. The injection of novel miRNA-2 agomir prevented the molting process, which led to high mortality. Ag, novel miRNA-2 agomir; NC, negative control.

Furthermore, the molting timepoint and molting rate were recorded. The results showed that the molting timepoint was significantly delayed to 19.23 ± 0.3944 days in the Ag-treated group compared with the NC group (15.47 ± 0.1417 days) (Student’s *t*-test, *P* < 0.0001). Moreover, the molting rate of the Ag-treated group was markedly decreased to 73.33% of the value obtained for the NC and blank control groups. These results strongly indicated that novel miRNA-2 overexpression exerts a negative effect on the molting process in *H. longicornis* nymphs (Figure 4).

### Novel miRNA-2 Depletion Results in Severe Molting Defects

To decipher the role of novel miRNA-2 in molting events, we blocked the expression of novel miRNA-2 by microinjecting Ant into *H. longicornis* nymphs at 5 days post blood meal ingestion and assessed the transcript and protein levels of CPR1 on day 2 after the microinjection. The qRT-PCR results showed that the mRNA expression level of CPR1 was not increased in the Ant-treated group (0.976 ± 0.05658) compared with the other groups, and no significant difference was observed between the NC (0.985 ± 0.05381) and blank control (0.973 ± 0.0771) groups. However, the Western blot analysis showed that the protein expression level of CPR1 was increased by Ant treatment compared with that found in the NC and blank control groups, which suggested that the blockage of novel miRNA-2 expression can upregulate CPR1 expression.

**FIGURE 5:**
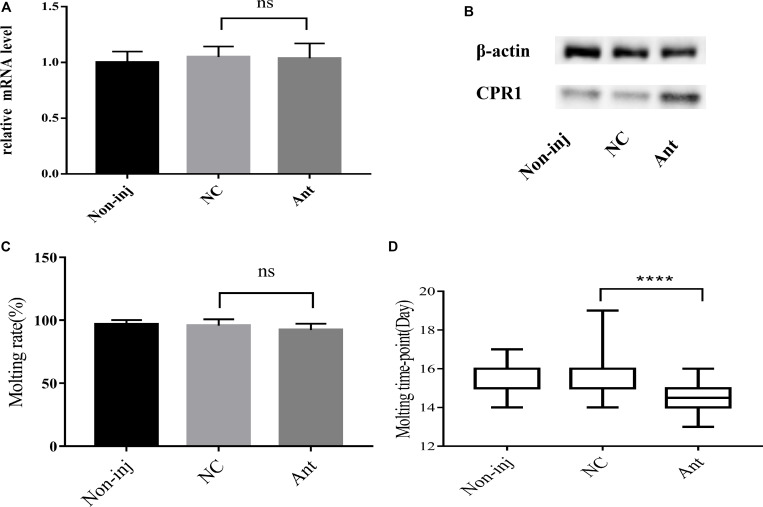
The blockade of novel miRNA-2 expression disrupted protein accumulation and impaired molt synchrony. **(A)** Transcription level of the target gene CPR1 after the inhibition of novel miRNA-2 expression. **(B)** Protein level of the target gene CPR1 after the inhibition of novel miRNA-2 expression. **(C)** Molting rate of nymphs injected with novel miRNA-2 Ant. **(D)** Molting timepoint of nymphs injected with novel miRNA-2 Ant. The data are shown as the means ± SEMs and were evaluated by Student’s *t*-test. ****P < 0.0001.

The molting timepoint of the Ant-treated group was reduced to 14.39 ± 0.1654 days compared with that of the NC group (15.4 ± 0.1633 days) (Student’s *t*-test, *P* < 0.0001). Moreover, the molting rate did not differ significantly between the Ant-treated and NC control groups (Figure 5).

**FIGURE 6:**
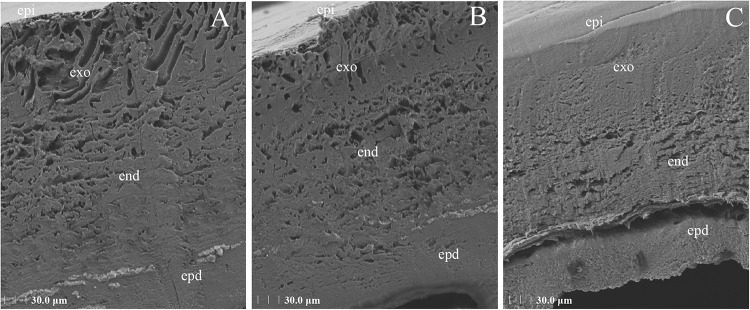
Ultrastructural architecture of the cuticle after injection of novel miRNA-2 antagomir. The tick nymph cuticle has three horizontal layers: the outermost layer is the epicuticle (epi), the second layer is the procuticle (pro), which contains two layers, namely, the exocuticle (exo) and the endocuticle (end), and the third layer is the epidermis (epd). In the NC and the blank control groups **(A,B)**, the tip of the microvilli-protruding portion was precipitated in the form of a cap, which was dispersed but tended to accumulate and aggregate. In the antagomir group **(C)**, these cap-like structures aggregated and formed a continuous layer, and clear cortical fusion occurred, resulting in a clear exuvial space.

The epidermal ultrastructure of the tick samples on day 2 after the microinjection was observed by scanning electron microscopy. The analysis of the NC and blank control groups showed that the tip of the microvilli-protruding portion was precipitated in the form of a cap, which was dispersed but tended to increase in number and aggregate. In the Ant group, these cap-like structures aggregated and formed a continuous layer, and clear cortical fusion occurred, which resulted in a clear exuvial space. These results showed that the blockade of novel miRNA-2 expression accelerated the molting process of *H. longicornis* nymphs (Figure 6).

### Effects of CPR1 RNA Interference (RNAi) on *H. longicornis* Nymph Molting Events

Because CPR1 was expressed in the epidermis and its expression changed during the normal molting process of *H. longicornis* nymphs, we examined the protein and mRNA expression of CPR1 and the molting timepoint and rate after CPR1 RNAi to further evaluate the potential roles of this gene. The mRNA expression of CPR1 were assessed on day 2 after iCut injection, and the results showed that the mRNA levels of CPR1 were significantly reduced in the iCut-treated group (0.725 ± 0.02172) compared with other group and not appreciably differ between the NC (0.996 ± 0.03407) and blank control (0.984 ± 0.02575) groups, Moreover, the Western blot analysis showed that the protein expression level of CPR1 was decreased by iCut treatment compared with that found in the NC and blank control groups, which suggested that a siRNA designed to target CPR1 successfully decreased the CPR1 transcript and protein levels in *H. longicornis* nymphs.

**FIGURE 7:**
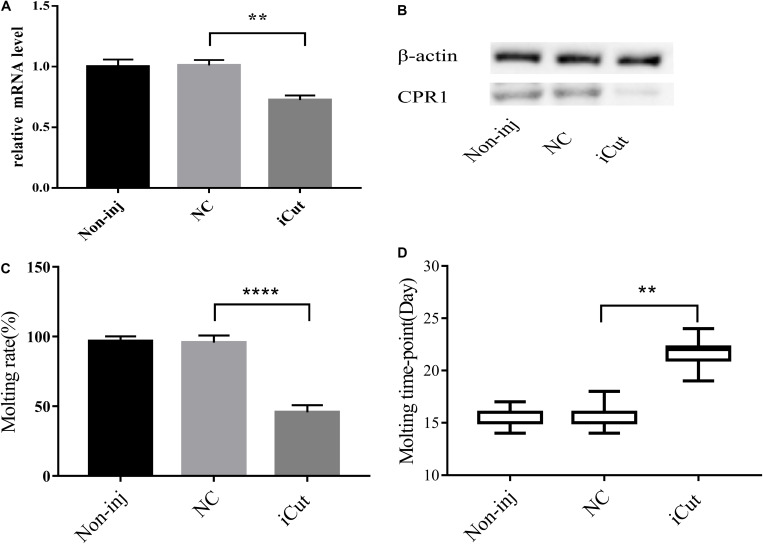
CPR1 RNAi significantly disrupted protein accumulation and delayed the molting timepoint. **(A)** Transcription level of the target gene CPR1 after the inhibition of CPR1. **(B)** Protein level of the target gene CPR1 after the inhibition of CPR1. **(C)** Molting rate of nymphs injected with CPR1-targeting siRNA. **(D)** Molting timepoint of nymphs injected with CPR1-targeting siRNA. The data are shown as the means ± SEMs and were evaluated by Student’s *t*-test. **P < 0.01 and ****P < 0.0001.

We also found that the molting rate of the iCut group was significantly decreased to 46.67% of the value obtained for the NC and blank control groups, whereas no significant difference was observed between the NC and blank control groups. Additionally, the molting timepoint was extended to 21.71 ± 0.3387 days in the iCut group compared with that in the NC group (15.5 ± 0.1216 days) (Student’s *t*-test, *P* < 0.0001), whereas no significant difference in the molting timepoint was observed between the NC and blank control groups (Figure 7).

### Phenotype Rescue Confirms That CPR1 Is a Direct Target of Novel miRNA-2 *in vivo*

To further verify whether CPR1 is an authentic target of novel miRNA-2 during *H. longicornis* nymph molting events, behavior phenotype rescue experiments were performed by microinjecting Ant for the blockage of novel miRNA-2 expression and using designed siRNA for the RNAi-mediated depletion of the CPR1 gene in *H. longicornis* nymphs at 5 days post blood meal ingestion. This method has been successfully used for validating miRNA mutant phenotypes in mosquitoes and ticks (Lucas et al., 2015; Hao et al., 2017).

**FIGURE 8:**
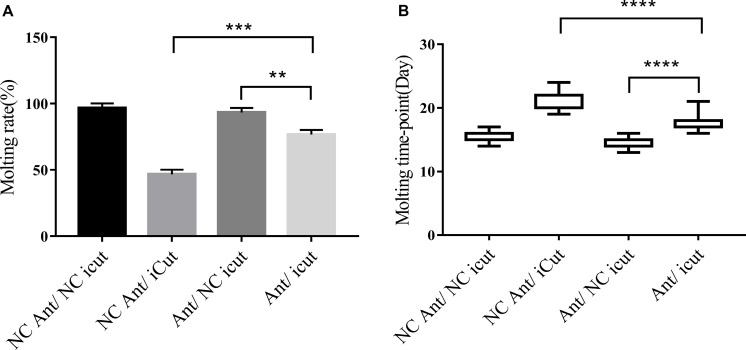
CPR1 RNAi partially rescued the novel miRNA-2-depletion phenotypes. **(A)** CPR1 RNAi improved the molting rate of nymphs treated with novel miRNA-2 antagomir. **(B)** CPR1 RNAi improved the molting timepoint of nymphs treated with novel miRNA-2 antagomir. The data are shown as the means ± SEMs and were evaluated by Student’s *t*-test. **P < 0.01, ***P < 0.001, and ****P < 0.0001.

The molting timepoint of the Ant/iCut coinjection group was significantly delayed to 17.95 ± 0.3104 days compared with that of the Ant/NC iCut group (14.32 ± 0.0171 days) (Student’s *t*-test, *P* < 0.0001), but this delay was shorter than that obtained for the iCut/NC Ant group (21.4 ± 0.3625 days) (Student’s *t*-test, *P* < 0.0001). However, the molting rate of the Ant/iCut group was notably declined to 76% of that obtained for the Ant group but was higher than that of the iCut group (46.67%) (Figure 8). These results suggested that the RNAi-mediated silencing of CPR1 resulted in severe phenotypes resulting from the inability of *H. longicornis* nymphs to undergo normal molting and, as expected, ameliorated the adverse phenotypes caused by the silencing of novel miRNA-2. Hence, the phenotypic rescue achieved by the administration of CPR1 RNAi to novel miRNA-2-depleted *H. longicornis* nymphs confirmed that CPR1 is an authentic target of novel miRNA-2 *in vivo*.

## Discussion

In this study, we first discovered and verified the presence of novel miRNA-2 in *H. longicornis*. The full-length novel miRNA-2 precursor has a length of 62 nt and exhibits a typical stem-loop structure, and its mature sequence is 22 nt in length. We named this miRNA hlo-miR-2.

hlo-miR-2 exhibits substantial stage and tissue specificity in *H. longicornis*. Specifically, it is highly abundant in unengorged larvae, unengorged nymphs and unengorged adults but not in eggs. This miRNA is also highly expressed in epidermal and midgut tissues compared with other tissues.

Our results identified CPR1 as a target gene of hlo-miR-2 and showed that this gene was also highly abundant in epidermal tissue. Moreover, the qRT-PCR analysis showed that the levels of hlo-miR-2 and CPR1 were negatively correlated during the molting process of *H. longicornis*.

This study provides the first determination of the full-length sequence of CPR1, which was 1171 bp in length. The CDS was 339 bp in length, and the binding site for hlo-miR-2 was located in the 3′ UTR of CPR1. The miRNA/mRNA fluorescence *in situ* hybridization results showed that CPR1 interacted directly with novel miRNA-2 in a spatially dependent manner.

The epidermis provides a barrier for the defense of ticks against adverse environmental changes and mechanical stimuli, and ticks must periodically shed and resynthesize their epidermis during development. Members of the CPR family, which is the largest family of CPs, are expressed in the procuticle and play a role in binding to chitin (Willis, 2010; Dittmer et al., 2015; Vannini et al., 2015; Arasteh et al., 2016). A simulation of the secondary structure of the protein indicated that this cuticular gene sequence contained a conserved chitin-binding 4 region, which is within a Rebers & Riddiford (R&R) motif and belongs to the CPR family (Tetreau et al., 2015).

As determined through dual luciferase reporter assay, the cotransfection of a luciferase reporter vector containing the *H. longicornis* CPR1 3′ UTR with the hlo-miR-2 mimic resulted in decreased *Renilla* luciferase activity *in vitro*, which suggested that CPR1 is a direct target gene of hlo-miR-2 *in vitro*.

The overexpression of hlo-miR-2 decreased CPR1 expression, significantly delayed the molting timepoint of *H. longicornis* nymphs, and markedly decreased the molting rate. The depletion of hlo-miR-2 in *H. longicornis* nymphs increased CP expression and accelerated the molting process in *H. longicornis* nymphs. These results suggested that hlo-miR-2 plays an important role in *H. longicornis* nymph molting events. The data obtained from the siRNA-mediated silencing of CPR1 during the *H. longicornis* nymph molting process suggested that CPR1 is necessary and sufficient for molting events.

The phenotypic rescue experiment showed that the siRNA/Ant group exhibited a delayed molting timepoint and a decreased molting rate compared with the Ant group, which indicated that the Ant can bind to hlo-miR-2 *in vivo* and counteract the inhibition of hlo-miR-2; thus, only the inhibitory effect of the CPR1 siRNA is reflected in the phenotype. Additionally, these results suggested that CPR1 significantly affects the formation of the original epidermis, whereas hlo-miR-2 has a significant inhibitory effect on the posttranslational level of CPR1, which indicated that CPR1 is a target gene of hlo-miR-2 *in vivo*.

A histological study showed that the *H. longicornis* 20-E titers increased sharply on day 6 after nymph engorgement, and this event was accompanied by the initiation of apolysis and the appearance of the exuvial space (Liu et al., 2001). Additionally, the same results were observed in *Ornithodoros moubata* and *Amblyomma hebraeum* (Diehl et al., 1982; Dotson et al., 1991). However, in this study, we found that hlo-miR-2 was maintained at a high level during the first 6 days after engorgement, and after this time, its level suddenly decreased and remained low until the end of the molting period. This trend is exactly opposite to those found for CPR1 transcription and the 20-E titer, which indicated that high hlo-miR-2 expression might inhibit molt initiation after *H. longicornis* nymph engorgement, but this possibility needs further study.

Previous studies of *Bombyx mori*, *Manduca sexta* and locusts have suggested that the level of *LmTwdl1*, a member of the Tweedle family of CPs, gradually increased beginning on day 5 after nymph engorgement, which is a vital period for cuticle renewal, and that this gene has conserved essential functions in molting events (Soares et al., 2011; Dittmer et al., 2015; Song et al., 2016). A study conducted by Liu et al. (2001) showed that deposition of the new epicuticle began on day 8 after nymph engorgement, which coincides with the time at which the 20-E titer peaked. However, in this study, the transcriptional level of CPR1 continued to increase from day 10 after engorgement until the molting process was completed. Furthermore, the silencing of CPR1 resulted in high mortality during the molting process before the next stage, which indicated that CPR1 plays a critical role during the late molting stage in *H. longicornis* nymphs.

## Conclusion

In conclusion, our study provides the first demonstration that hlo-miR-2 is an authentic miRNA in *H. longicornis* that exhibits significant tissue and temporal specificity. The results from RNAi experiments showed that CPR1 is highly associated with the molting process in *H. longicornis* nymphs. More importantly, through dual luciferase reporter assays and phenotypic rescue experiments, we uncovered the role of hlo-miR-2 in molting events in *H. longicornis* nymphs. Furthermore, we identified CPR1 as a direct target of hlo-miR-2, and the obtained functional evidence verified that the hlo-miR-2-mediated regulation of CPR1 is essential for molting development in *H. longicornis* nymphs. In summary, our research indicates that miRNAs constitute a novel important regulator of the molting process in addition to hormones. The functional evidence implicating CPR1 in molting events contributes to a better understanding of the distinct functions of the CPR family in ticks and will aid the development of the promising application of cuticular RNAi in tick control.

## Data Availability Statement

The datasets generated or analyzed during this study are included in this published article and its additional files.

## Ethics Statement

The animal study was reviewed and approved by Approval No. LVRIAEC2012-011.

## Author Contributions

G-YL and W-GL designed the experiments. W-GL, Z-QQ, JL, Q-YR, Z-GW, JN, Y-CT, X-FQ, X-FX, H-LL, R-HX, and R-GC performed the experiments. W-GL analyzed the data and wrote the manuscript. G-YL, Z-QY, J-XL, SX, MR, HW, and HY critically revised the manuscript. All the authors read and approved the final version of the manuscript.

## Conflict of Interest

The authors declare that the research was conducted in the absence of any commercial or financial relationships that could be construed as a potential conflict of interest.

## Footnotes

1 http://rna.tbi.univie.ac.at/cgi-bin/RNAWebSuite/RNAfold.cgi

2 https://bibiserv.cebitec.uni-bielefeld.de/rnahybrid/

3 https://genie.weizmann.ac.il/pubs/mir07/mir07_data.html

4 http://www.microrna.org/microrna/home.do

## Abbreviations

Ag: agomir
Ant: antagomir
cDNA: complementary deoxyribonucleic acid
CDS: coding sequence
CPR: cuticular protein with Rebers and Riddiford consensus
CPs: cuticular proteins
Cy3: sulfo-cyanine 3
DAPI: 2-(4-amidinophenyl)-6-indolecarbamidine dihydrochloride
DMEM: Dulbecco’s modified Eagle medium
DNA: deoxyribonucleic acid
FAM: carboxyfluorescein
FBS: fetal bovine serum
HRP: horseradish peroxidase
iCut: interference RNA for CPR1
miRNA: micro-ribonucleic acid
NC: negative control
PBS: phosphate buffered saline
PVDE: polyvinylidene fluoride
qRT-PCR: quantitative real-time PCR
qRT-PCR: quantitative reverse transcription polymerase chain reaction
RNA: ribonucleic acid
RNAi: RNA interference
SDS-PAGE: sodium dodecyl sulfate polyacrylamide gel electrophoresis
TBST: Tris-buffered saline Tween-20
USA: United States of America
UTRs: untranslated regions.
